# Is it Possible to Obtain Immunofluorescence Data in Formalin-Fixed Paraffin-Embedded Skin Samples for the Diagnosis of Pemphigus Vulgaris and Bullous Pemphigoid

**DOI:** 10.5146/tjpath.2023.01607

**Published:** 2024-01-22

**Authors:** Deniz Ates Ozdemir, Ozay Gokoz, Arzu Saglam

**Affiliations:** Department of Pathology, Hacettepe University, Faculty of Medicine, Ankara, Turkey

**Keywords:** Protease digestion, IgG, Pemphigus vulgaris, Bullous pemphigoid, C4d

## Abstract

***Objective: *** The gold-standard method for assessment of autoimmune bullous disease is direct/indirect immunofluorescence (IF) examination applied to fresh frozen tissue. Since the sensitivity of IF is greatly reduced in formalin-fixed paraffin-embedded (FFPE) tissues, IF cannot be relied upon in these samples. However, immunohistochemistry with the C4d antibody is a promising marker used as a surrogate for immune complex deposition, in nephropathology practice, and the paraffin IF method is also used as a “salvage” technique when fresh frozen tissue is not available or lacks glomeruli. We aimed to investigate whether it is possible to obtain immunofluorescence data from FFPE tissues diagnosed with bullous pemphigoid (BP) and pemphigus vulgaris (PV) and its relationship with inflammatory parameters in the skin.

***Material and Methods:*** Eighty-nine in-house cases with both IgG and C3 positivity by routine immunofluorescence examination were included in the study. Inflammation parameters were evaluated in hematoxylin-eosin sections. Immunofluorescence study with IgG protease digestion and C4d immunohistochemistry were performed.

***Results: *** Results of 83 biopsies were obtained by paraffin immunofluorescence with IgG. There were positive reactions in 28 (34%) of these 83 biopsies. Five of the 28 positive results belonged to BP (18%), and 23 were PV (82%). Ten positive results were on lesional skin (36%), and 18 (64%) were on non-lesional skin. In the immunohistochemical study with C4d, 84 biopsy results were obtained. There were positive reactions in 34 (40.4%) of 84 biopsies. Of the 34 positive results, 12 belonged to BP (35.3%) and 22 to PV (64.7%). Again, 22 (64.7%) of 34 positive results belonged to lesional skin, and 12 (35.3%) belonged to non-lesional skin. When both techniques were used together, 44 (54%) of 81 biopsies yielded positive results for at least one of the two studies, while in 37 (46%), both tests showed negative results.

***Conclusion:*** The sensitivity of both IgG and C4d was less than in the literature, especially in BP-diagnosed biopsies. Positive samples were mostly PV. In conclusion, obtaining immunofluorescence data in FFPE samples is possible and is independent of the related skin being lesional or not, however, negative results should not be relied upon.

## INTRODUCTION

Diagnosing an autoimmune bullous disease is a challenging field of dermatology and dermatopathology. Light microscopic findings are insufficient for accurate classification and additional direct immunofluorescence (IF) findings are necessary. According to the EADV (European Academy of Dermatology and Venereology), a total of 2 skin biopsies are needed if an autoimmune bullous disease is suspected: one from the lesional skin and another from the perilesional intact/undamaged skin (1). The sample taken from the lesional tissue should be sent to the pathology laboratory in formaldehyde solution. The other biopsy is taken for direct IF examination and should be delivered to the pathology laboratory fresh. Michel’s solution, which is an expensive solution, can also be used as a transport solution for tissues that cannot be sent immediately (2). Paraffin tissue processing procedures are applied to the sample sent in formaldehyde, and the sections are stained with hematoxylin-eosin.

In recent years, it has been reported that paraffin immunofluorescence (PIF) examination can be performed with various special protocols in cases where immunofluorescence examination cannot be performed or when there is insufficient glomerulus in nephropathology practice; this method has been recognized as a “salvage method” in nephropathology (3,4). In skin pathology, Valencia-Guerrero et al.’s study in 2018 is the only study published on PIF in autoimmune bullous diseases (5). This study has demonstrated that this technique is valuable, though less sensitive than routine frozen examination. Another method that indirectly indicates the presence of immune complexes in formalin-fixed paraffin-embedded (FFPE) skin tissues is immunohistochemical studies with C4d antibody, an indicator of classical pathway-related complement activation. Studies have shown that demonstration of C4d is successful in aiding the diagnosis (6–10).

In daily practice, when the histopathologist suspects autoimmune bullous disease in hematoxylin-eosin sections, disease classification cannot be made and a new biopsy is requested. Furthermore, there can be rare instances where paraffin processing is inadvertently applied to tissues sent for DIF examination, or the lesional skin is biopsied, and these may cause delays in diagnosis and possible morbidity.

In this study, we aimed to perform a PIF study with the protease digestion method by using IgG and standard C4d immunohistochemistry in formalin-fixed paraffin-embedded lesional and non-lesional skin biopsies with pemphigus vulgaris and bullous pemphigoid diagnoses, and to assess the effect of inflammation parameters (since the preference for biopsy is non-inflamed perilesional skin in routine practice, we wanted to investigate the relationship of inflammation parameters such as inflammatory cells, fibrin, presence or absence of acantholysis, and dominant inflammatory cell type).

## MATERIAL and METHODS

**Figure 1:**
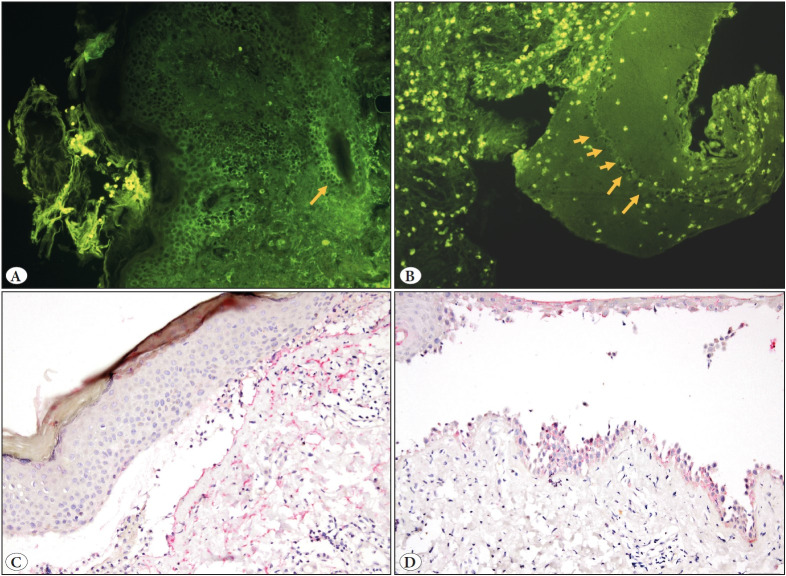
**A)** In the biopsy of pemphigus vulgaris, an intraepidermal positive reaction with IgG via protease digestion is seen in the lesioned skin. An intraepidermal positive reaction was observed in hair follicle epithelium (Orange arrow) (DIF-P, 100x). **B)** In a case of bullous pemphigoid, positive reaction with IgG by protease digestion is seen along the basement membrane in the roof of the split cavity (Orange arrows) (DIF-P, 100x) Autofluorescent cells are leukocytes. **C)** In a case of bullous pemphigoid, a positive reaction is seen along the basement membrane with C4d (C4d, 100x). **D)** Positive reaction with C4d along the basement membrane in a case of bullous pemphigoid (C4d, 100x)

The research was approved by the Institutional Research Board (GO-1882 /25-9-2018). The research complied with the Helsinki Declaration. A total of 89 in-house (37 non-lesional, 52 lesional) biopsies of 43 patients diagnosed with pemphigus vulgaris and bullous pemphigoid based on clinical-pathological correlation between 2014 and 2018 were included in the study. All cases had deposition of IgG and C3 as demonstrated with routine direct immunofluorescent microscopy applied to frozen sections. Three unstained 5µ sections were obtained from the FFPE tissue samples of these cases. One of the unstained slides was stained with H&E to determine microscopic parameters. These microscopic parameters were classified in regards to the presence or absence of inflammation: whether inflammation was present, its severity (assessed as mild, moderate, and intense), and the dominant cell type (indeterminate-dominant cell undetectable, eosinophils, lymphocytes, and neutrophils). Acantholysis and the presence or absence of fibrin deposition were noted. The second unstained section was used for the PIF study using the proteinase digestion method as detailed. Sections were kept in phosphate salt buffer (PBS) for 10 minutes after deparaffinization and rehydration. They were then treated with 0.05% proteinase (Sigma cat # P8038) in PBS solution at 37°C for 5 minutes for antigen expression. Then, the IgG antibody labeled with FITC (fluorescent isothiocyanide) was dripped onto the sections and kept in the dark for 45 minutes. Sections were washed two times with PBS and covered with immunofluorescence-specific mounting medium (Dako; cat. #S3023). The last section was stained with C4d immunohistochemistry in the Leica Bond Max (Shandon, Frankfurt, Germany) autostainer with appropriate positive and negative controls as detailed. After exposure to EDTA, sections were incubated for 30 minutes with the primary antibody C4d (404A-15, Rabbit polyclonal antibody, 1/200 dilution, Cell Marque, Rocklyn, CA). 3-amino-9-ethylcarbazole (AEC-Red) chromogen was used. Selective linear staining/deposition along the dermo-epidermal junction was accepted as positive for samples diagnosed with bullous pemphigoid, and intraepidermal membranous staining/deposition was accepted as positive for samples diagnosed with pemphigus vulgaris (Figure 1). Two experienced dermatopathologists (OG, DAO) performed fluorescent microscope evaluation. Cases with discordant findings were re-evaluated jointly, and a consensus was reached.

The Shapiro-Wilk test, histogram, box-line, and Q-Q graph results were used to determine the conformity of continuous variables (age) to normal distribution. The difference between the two independent groups (age) was compared with the Mann-Whitney U test for continuous variables. The difference between groups was examined for categorical variables, with the Chi-Square or Fisher Exact test (gender, IgG, C4d positivity or negativity, presence or absence of acantholysis, inflammatory cells, fibrin, predominant cell type, lesional / non-lesional, PV/BP diagnosis). The significance level was taken as 0.05 in all tests. Statistical analyses were performed using IBM SPSS version 23 (IBM Co. USA).

## RESULTS

A total of 89 biopsies of 43 patients with positive IgG and C3 immunofluorescence results applied to frozen sections with clinicopathological correlations were accessed from the hospital’s patient file software. Of the 43 patients, 21 were male, and 22 were female. Their mean age was 60.70 years (youngest: 27, oldest: 91 years, standard deviation 18.79). Twenty-four of the 43 patients were diagnosed with pemphigus vulgaris (PV), and 19 were diagnosed with bullous pemphigoid (BP). Forty-seven biopsies were from patients diagnosed with PV and 42 with BP. Of the 89 biopsies, 52 were lesional, and 37 were non-lesional skin (Table 1).

**Table 1:** Number of lesional and non-lesional biopsies by diagnosis

	Lesional*	Non-lesional**
**BP (42 biopsies)**	22	20
**PV (47 biopsies)**	30	17
**Total**	52	37

**BP:** Bullous pemphigoid diagnosed samples, **PV:** Pemphigus vulgaris diagnosed samples. *Lesional skin refers to skin with a dermatologic lesion and microscopic findings specific to the disease (PV/BP), **Non-lesional skin refers to skin tissue without a dermatologic lesion (usually representing the perilesional area biopsied for immunofluorescence evaluation.

The dominant cell type of BP biopsies (42) was lymphocytes in 6 (14%), eosinophils in 13 (31%), and neutrophils in 4 (10%). In 19 (45%), the presence of a dominant cell was not detected. Of the PV biopsies (47), the predominant cell type was lymphocytes in 22 (47%), eosinophils in 4 (9%), and neutrophils in 1 (2%). In 20 (42%), the presence of a dominant cell was not detected.

The relationship between IgG and C4d results and the histomorphologic parameters commonly seen in autoimmune bullous diseases is shown in detail in Table 2, Table 3, and Table 4.

**Table 2:** The relationship between IgG results with histomorphologic parameters commonly seen in autoimmune bullous diseases

	**Lesion**	**Inflammation**	**Acantholysis**	**Fibrin**	**Diagnosis**
**IgG Negative** **(55 biopsy)**	No: 23 (42%)	No: 22 (40%)	No: 40 (73%)	No: 30 (55%)	PV: 21 (38%)
	Yes: 32 (58%)	Yes: 33 (60%)	Yes: 15 (27%)	Yes: 25 (46%)	BP: 34 (62%)
**IgG Positive** **(28 biopsy)**	No: 10 (36%)	No: 12 (43%)	No: 13 (47%)	No: 23 (82.1%)	PV: 23 (82%)
	Yes: 18 (64%)	Yes: 16 (57%)	Yes: 15 (54%)	Yes: 5 (18%)	BP: 5 (18%)
**p Value**	0.289	0.802	0.018*	0.013*	0.000*

*p<0.005 were considered statistically significant. **BP:** Bullous pemphigoid, **PV:** Pemphigus vulgaris.

### a) Results of Paraffin Immunofluorescence with IgG

Data were obtained from 83 of the 89 biopsies due to drop-out during the immunofluorescence study and the absence of epidermis in the resulting sections. There was positive staining in 28 (34%) of the 83 biopsies. Of the 28 specimens positive with PIF IgG, 5 belonged to BP (18%) and 23 to PV (82%). There were 10 (36%) that were lesional skin, 18 (64%) were non-lesional, 16 had inflammation, and 5 contained fibrin (Table 2 and Table 3).

**Table 3:** IgG and C4d results of biopsies by diagnosis

	**IgG**	**C4d**
BP*	Positive: 5 (13%)	Positive: 12 (32%)
	Negative: 34 (87%)	Negative: 26 (68%)
PV**	Positive: 23 (53%)	Positive: 22 (48%)
	Negative: 21 (48%)	Negative: 24 (52%)
**Total**	83	84

*Results were obtained from 39 BP biopsies with IgG and 38 BP biopsies with C4d, **Results were obtained from 44 PV biopsies with IgG and 46 PV biopsies with C4d. **BP:** Bullous pemphigoid diagnosed samples, **PV:** Pemphigus vulgaris diagnosed samples.

Amongst the 55 IgG-negative specimens, 32 were lesional, 22 had inflammation, 40 showed acantholysis, 25 contained fibrin, and 34 (62%) were samples diagnosed as bullous pemphigoid (Table 2).

When specimens with positive and negative PIF IgG were compared, statistically significant differences were obtained with regard to the presence of acantholysis (present in 72% of negative samples) and fibrin (no fibrin in 82% of positive samples), and the diagnosis of the disease (82% of positive samples, PV). (p values 0.018 - 0.013 - 0.000, respectively). Age and biopsy localization did not yield significant results in terms of positivity and negativity.

### b) Results of Immunohistochemistry with C4d

Data were obtained from 84 of 89 biopsies due to drop-out during the immunofluorescence study and the absence of epidermis in the resulting sections. There was positive staining in 34 (40%) of 84 biopsies. Of the 34 specimens positive for C4d, 12 were BP- (35%) and 22 (65%) PV-diagnosed biopsies. There were 22 (65%) that were lesional, and 12 (35%) were non-lesional. Twenty samples had inflammation, and 8 contained fibrin (Table 4).

**Table 4:** The relationship between C4d results and histomorphologic parameters commonly seen in autoimmune bullous diseases

	**Lesion**	**Inflammation**	**Acantholysis**	**Fibrin**	**Diagnosis**
**C4d Negative** **(50 biopsies)**	No: 23 (46%)	No: 22 (44%)	No: 36 (72%)	No: 30 (60%)	PV: 24 (48%)
	Yes: 27 (54%)	Yes: 28 (56%)	Yes: 14 (28%)	Yes: 20 (40%)	BP: 26 (52%)
**C4d Positive** **(34 biopsies)**	No: 12 (35%)	No: 14 (41%)	No: 17 (50%)	No: 26 (77%)	PV: 22 (65%)
	Yes: 22 (65%)	Yes: 20 (59%)	Yes: 17 (50%)	Yes: 8 (24%)	BP: 12 (35%)
**p Value**	0.329	0.797	0.04*	0.116	0.131

*p<0.005 was considered statistically significant. **BP:** Bullous pemphigoid diagnosed samples, **PV:** Pemphigus vulgaris diagnosed samples.

Of the 50 C4d negative specimens, 27 were lesional, 28 had inflammation, 14 showed acantholysis, 20 contained fibrin, and 26 (52%) were samples diagnosed as bullous pemphigoid. These results are summarized in Table 3 and Table 4.

There was a statistically positive correlation between positive C4d staining and the presence of acantholysis (p=0.04). Age and biopsy localization did not yield significant results regarding C4d positivity and negativity.

## DISCUSSION

The paraffin immunofluorescence (PIF) method has been used as a salvage technique in nephropathology for quite some time (3,4). However, its use in skin pathology has only been reported once (5). In this study, PIF for the diagnosis of autoimmune bullous disease was performed on samples representing non-lesional skin. In the same study, 14 of 18 BP cases (78%) and 3 of 7 pemphigus cases (43%) were positive. In our study, IgG PIF was applied to lesional and non-lesional skin. “Lesional skin” refers to skin with a dermatologic lesion and microscopic findings specific to the disease (PV/BP), such as inflammation and a split. “Non-lesional skin” refers to tissue without a dermatologic lesion (usually representing the perilesional area biopsied for immunofluorescence evaluation) and microscopically without features such as inflammation or a split. A positive reaction was obtained in 5 of 39 BP biopsies (13%) and 23 of 44 PV biopsies (53%). Cases diagnosed with PV comprised most of our cases that showed IgG PIF positivity (23/28, 82%), with cases of BP comprising the minority (5/28 positive biopsy).

In contrast to the study mentioned above, false negative results in our series were predominantly seen in BP-diagnosed biopsies. It was observed that 10 (36%) of the 28 positive results belonged to the lesional skin and 18 (64%) to non-lesional skin. Although most positive biopsies belong to non-lesional skin, there was no statistically significant difference in PIF IgG positivity between lesional and non-lesional skin or the presence or absence of inflammation (Table 1). Our findings highlight the possibility of demonstrating IgG deposition with PIF in lesional skin biopsies. Although the sensitivity is low, this finding opens up the opportunity to diagnose bullous diseases from a single biopsy in those patients where it is the histopathologist who suspects an autoimmune bullous disease or the biopsy of the accompanying non-lesional skin is mistreated or harmed.

Regarding C4d immunohistochemistry, 12 out of 48 BP biopsies were positive (25% of all BP-diagnosed biopsies). We also found that 22 of 46 PV-diagnosed samples were positive (48% of all PV-diagnosed biopsies). In the literature, we usually found more promising results than ours when we reviewed the positivity of C4d immunohistochemistry in BP and pemphigus. In series with the number of BP cases ranging between 4 and 30, the positivity rate with C4d immunohistochemistry in BP varied between 77% and 90% (7–10). On the other hand, in the study by Margo and Dyrsen, 4 of 17 BP cases (23%) had a low rate of positive results, just like us (6).

Our study is the series with the highest number of biopsies since it included 42 BP biopsies. Regarding PV, C4d immunohistochemistry results in FFPE tissues were obtained in two series with 11 and 22 pemphigus cases (6,8). In these series, C4d immunohistochemistry positivity rates in FFPE tissues were 82% (6) and 77% (8). Both of these studies reached a higher positivity rate than ours. This difference may be due to the variable levels of autoantibodies targeting structural proteins and the associated difference in the amount of immune complex deposition. Serologic titers of the relevant autoantibodies need to be known and compared to understand this. Also, intensity comparisons in direct immunofluorescence studies are needed to reflect the amount of immunocomplex deposition.

The melanin pigment content in keratinocytes is a factor that complicates evaluation, especially in the intraepidermal area. Unlike other studies, we performed C4d immunohistochemistry with AEC (3-Amino-9-ethylcarbazole) chromogen, which allowed for an accurate evaluation. The presence of acantholysis showed a statistically significant correlation with C4d positivity (72% of negative biopsies did not have acantholysis). That may be because positive biopsies are mostly PV-diagnosed samples (64%), and acantholysis is a hallmark histologic feature of PV. It could also be that complement activation is more prominent in such cases. Statistically, whether the tissue was lesional or non-lesional did not affect the result. Similar to PIF IgG, it can be concluded that diagnosis of bullous diseases can be made possible with C4d immunohistochemistry from a single lesional biopsy, although it has low sensitivity.

Furthermore, if PIF IgG and immunohistochemical C4d evaluation are used together in a biopsy, our findings suggest that at least one can be positive in 54% of the biopsies. Combined with the literature data, our study shows that the positivity rate may be much lower.

Is performing C4d immunohistochemistry and/or IgG PIF on formalin-fixed paraffin-embedded tissues worthwhile? There is no straightforward answer to this question. When performed together, 54% of the biopsies were positive in our series. That is like flipping a coin. If we get a positive biopsy, we save the patient from another punch biopsy. The question may be whether we want to save the patient from a new punch biopsy, which is the gold standard? If it will cause morbidity (cosmetic problem, general impairment in a patient that is not suitable for biopsy, pediatric patient, delicate areas such as conjunctiva) or if the patient does not allow a biopsy, the answer is yes, we do.

In summary, our findings suggest that PIF IgG can also be used as a salvage technique in skin biopsies of patients with bullous diseases, similar to kidney biopsies, and reveal the fact that even biopsies of lesional skin can potentially be diagnostically useful, a unique finding that has not been previously mentioned in the literature. However, one should remember that the absence of staining should not be relied upon to rule out bullous disease.

## Conflict of Interest

We have no conflicts of interest to disclose.

## Ethical Approval

This project was approved by the Hacettepe University non-interventional studies ethics committee with decision number 18/882-25.
